# Complete Genome Analysis of *Subtercola* sp. PAMC28395: Genomic Insights into Its Potential Role for Cold Adaptation and Biotechnological Applications

**DOI:** 10.3390/microorganisms11061480

**Published:** 2023-06-01

**Authors:** Ryoichi Yamada, So-Ra Han, Hyun Park, Tae-Jin Oh

**Affiliations:** 1 Department of Life Science and Biochemical Engineering, SunMoon University, Asan 31460, Republic of Korea; tarkahr825@sunmoon.ac.kr (R.Y.); 553sora@sunmoon.ac.kr (S.-R.H.); 2 Bio Big Data-Based Chungnam Smart Clean Research Leader Training Program, SunMoon University, Asan 31460, Republic of Korea; 3 Genome-Based BioIT Convergence Institute, Asan 31460, Republic of Korea; 4 Division of Biotechnology, College of Life Science and Biotechnology, Korea University, Seoul 02841, Republic of Korea; hpark@korea.ac.kr; 5 Department of Pharmaceutical Engineering and Biotechnology, SunMoon University, Asan 31460, Republic of Korea

**Keywords:** *Subtercola* sp., complete genome, CAZyme, cold-adapted bacteria, polysaccharide

## Abstract

This study reports the complete genome sequence of *Subtercola* sp. PAMC28395, a strain isolated from cryoconite in Uganda. This strain possesses several active carbohydrate-active enzyme (CAZyme) genes involved in glycogen and trehalose metabolism. Additionally, two specific genes associated with α-galactosidase (GH36) and bacterial alpha-1,2-mannosidase (GH92) were identified in this strain. The presence of these genes indicates the likelihood that they can be expressed, enabling the strain to break down specific polysaccharides derived from plants or the shells of nearby crabs. The authors performed a comparative analysis of CAZyme patterns and biosynthetic gene clusters (BGCs) in several *Subtercola* strains and provided annotations describing the unique characteristics of these strains. The comparative analysis of BGCs revealed that four strains, including PAMC28395, have oligosaccharide BGCs, and we confirmed that the pentose phosphate pathway was configured perfectly in the genome of PAMC28395, which may be associated with adaptation to low temperatures. Additionally, all strains contained antibiotic resistance genes, indicating a complex self-resistance system. These results suggest that PAMC28395 can adapt quickly to the cold environment and produce energy autonomously. This study provides valuable information on novel functional enzymes, particularly CAZymes, that operate at low temperatures and can be used for biotechnological applications and fundamental research purposes.

## 1. Introduction

Although glaciers are generally thought to only appear in cold environments, glaciers also exist in equatorial Africa, such as on Mt. Kilimanjaro in Tanzania, Mt. Kenya in Kenya, and the Rwenzori Mountains in Uganda [1,2]. Among them, those in the Rwenzori Mountains in Uganda are expected to disappear soon. However, research on this region is insufficient [3]. Tropical mountain environments face the imminent threat of disappearing due to climate warming, reduced humidity, and diminished cloud cover. The ongoing global warming trend suggests that these regions will experience substantial damage, second only to the Arctic, as the Earth’s climate continues to warm [4]. Thus, continued interest in tropical mountain environments and research about their ecosystems, such as restoration mechanisms, are needed to reduce damage.

Although glacier regions have sub-zero temperatures and harsh environments, they are home to several microbes that are significant sources of cold-adapted microorganisms [5]. Microorganisms isolated from extreme environments can adapt to and thrive in various environmental conditions, including freeze–thaw cycles, nutrient limitations, high salinity, and low temperatures [6]. Isolating microorganisms from extreme environments can offer valuable insights into their unique genomic characteristics, ability to produce various cold-adapted enzymes, and genetic adaptability. At lower temperatures in environments such as the Arctic and Antarctic, oxygen solubility increases, leading to higher levels of reactive oxygen species (ROS) and the resulting oxidative stress. While bacterial adjustment pathways involving the production of ROS have been recognized, the molecular mechanisms underlying these pathways and alternative metabolic pathways in cold environments remain incompletely understood [7]. Cold-adapted microorganisms contain significant amounts of polysaccharides such as glycogen, trehalose, and maltodextrin as essential carbohydrates. Bacteria with complete metabolic pathways have demonstrated their ability to conserve and efficiently utilize energy in these conditions. Carbohydrate-active enzymes (CAZymes) might also play a crucial role in the survival of microorganisms at freezing temperatures [8].

A comparative genomic analysis based on genome data of available bacteria strains could reveal common species characteristics of large populations and the physiological characteristics of bacteria. Moreover, some research studies on the characteristics of related bacteria have proven that unique functional genes might be acquired from each natural environment and enhance their potential applications, such as in bioremediation, biomedical, and healthcare contexts [9]. The *Subtercola* genus, a member of the family *Microbacteriaceae*, belongs to the phylum Actinomycetota. The use of *Subtercola boreus* strain DSM13056^T^ as a new psychrophilic actinobacterium isolated from groundwater was first mentioned in 2000 [10]. *Subtercola* species isolated from cold environments such as high-altitude extreme cold environmental niches, including snowy mountains [11], cold volcano lakes [12], glacier cryoconite [13], and plants from cold environments [14], have been reported. In addition, through sequence similarity analysis of the 16S rRNA gene and metagenomics analysis (more than 96%), it is possible to identify species related to the *Subtercola* genus in cold environments, such as the cold habitats of Antarctic and Arctic Oceans. These results show indication of a species of *Psychrophiles* [15,16,17]. Currently, various researchers have performed systematic research on cold environments in mid-latitudes and polar areas such as the Arctic and Antarctic, and some results have been obtained through the study of the cold environment [8]. The *Subtercola* genus is still considered new. So far, few studies have performed comparative genomic analysis of this genus. *Subtercola* species are a relatively established genus within the family *Microbacteriaceae*. It currently contains five recognized members: S. *boreus*, S. *frigoramans*, S. *lobariae*, S. *vilae*, and S. *endophyticus*. Genome sequences of 16 strains belonging to the *Subtercola* species have been registered in the National Center for Biotechnology Information (NCBI) database. Moreover, Jiang et al. [14] recently discovered a new species within the *Subtercola* genus. Strain PAMC28395 was obtained from a glacier located in the Rwenzori Mountains, and this study presents the complete genome sequence of this strain. A comparative genomic analysis was conducted using complete genome data in the NCBI database to gain insights into its characteristics within the *Subtercola* genus. This analysis helped predict gene functions and provided a deeper understanding of the mechanisms by which PAMC28395 adapts to its natural environment, uncovering emerging evolutionary characteristics. This study’s comprehensive examination of complete genomes offers valuable insights into the evolution and genetic composition of various strains and species inhabiting cold environments.

## 2. Materials and Methods

### 2.1. Isolated Bacteria and DNA Extraction

The *Subtercola* sp. PAMC28395 strain was isolated from glaciers of Mt. Rwenzori, Uganda, using 0.001 × TSB Gellan gum. This strain was isolated at 10 °C. It grew at temperatures of up to 15 °C using a pure Tryptic Soy Broth (TSB, Difco Laboratories Inc., Franklin Lakes, NJ, USA) and produced enough cells for extraction of genomic DNA. The genomic DNA of PAMC28395 was extracted using a QIAamp DNA Mini Kit (Qiagen Inc., Valencia, CA, USA) according to the manufacturer’s protocol. Purified genomic DNA was quantified with a spectrophotometer (Biochrome, Libra S35PC, Cambridge, UK). In addition, the quality of the extracted DNA was determined based on A260/A280 and agarose gel e We have checked and provided sharper figurelectrophoresis before performing further research.

### 2.2. Genome Sequencing and Assembly Process

Before the sequencing process, quantity and purity were determined using an Agilent 2100 Bioanalyzer (Agilent Technologies, Santa Clara, CA, USA) to obtain complete genome sequencing. Genome sequencing was performed using PacBio RS II single-molecule real-time (SMRT) sequencing technology (Pacific Biosciences, Menlo Park, CA, USA). SMRTbell library inserts (20 kb) were sequenced using SMRT cells. Raw sequence data were generated and de novo assembled using the hierarchical genome assembly process (HGAP) protocol [18] and RS HGAP4 Assembly in SMRT analysis software (ver. 2.3; Pacific Biosciences, SMRT Link 4.0.0) protocols. Annotation was performed with the NCBI Prokaryotic Genome Annotation Pipeline (PGAP). Information about PGAP can be found here: https://www.ncbi.nlm.nih.gov/genome/annotation_prok/). In addition, coding DNA sequences (CDSs) were predicted and annotation was performed with a Rapid Annotation using Subsystem Technology (RAST) server (https://rast.nmpdr.org/) [19]. A circular map of the PAMC28395 strain’s complete genome was visualized using the CGView server in Proksee (https://proksee.ca/) [20,21].

### 2.3. Phylogenetic Tree Analysis to Identify Species

The PAMC28395 strain was compared with diverse gena in Microbacteriaceae using 16S rRNA phylogenetic analysis. Alignments were performed using the NCBI database’s Basic Local Alignment Search Tool and analyzed using EzBioCloud (www.ezbiocloud.com). Finally, 16S rRNA sequences were aligned using MUSCLE [22,23] to reconstruct a phylogenetic tree using neighbor-joining maximum likelihood with 1000 bootstrap replications and similarity sequences through MEGA X [24].

### 2.4. Average Nucleotide Identity (ANI) and DNA–DNA Hybridization (dDDH)

We downloaded whole genome sequences of *Subtercola* species available in GenBank (https://www.ncbi.nlm.nih.gov). We then determined the relationship of PAMC28395 with other strains from the same species and confirmed their similarity by comparing values of ANI calculated using an OrthoANI in OAT (the Orthologous Average Nucleotide Identity Tool) [25]. In addition, digital values of dDDH were calculated using the Genome-to-Genome Distance Calculator (GGDC) 3.0 provided by the Leibniz Institute DSMZ website (https://ggdc.dsmz.de/ggdc_background.php) with recommended parameters and/or default settings [26].

### 2.5. Annotation Gene to Genomic Features of Subtercola sp. PAMC28395

We used diverse annotation tools to survey the genomic features of PAMC28395. First, the genome of strain PAMC28395 was annotated using the RAST server. Second, predicted gene sequences were translated and searched in the NCBI non-redundant database. Next, we obtained functional annotation genes, including Clusters of Orthologous Genes (COGs) from EGGNOG 5.0 [27] and function genes and related pathways in the Kyoto Encyclopedia of Genes and Genomes (KEGG) using a cut-off value of 0.01 [28]. Moreover, we tried to predict the CAZyme. CAZyme analyses were conducted by running the dbCAN3 server (https://bcb.unl.edu/dbCAN2/) [29] with an e-value cut-off of 1e-15 and a coverage >0.35. In addition, we used DIAMOND [30] (e-value < 1e-102) and eCAMI [31] to improve prediction accuracy. Finally, biosynthetic gene clusters (BGCs) and putative BGCs encoding secondary metabolites were identified using antiSMASH bacterial version 6.1.1 [32] with default parameters. All prediction features and antibiotic resistance genes in the bacterial genome were selected and evaluated with the Antibiotic Resistance Target Seeker (ARTS) version 2.0 [33] web server (https://arts.ziemertlab.com/).

## 3. Results and Discussion

### 3.1. Complete Genome Information of Subtercola sp. PAMC28395

As shown in Table 1, the complete genome of PAMC28395 comprises a circular chromosome of 3,214,492 bp with a GC content of 64.5%. The number of contigs was one. A total of 2930 genes were predicted on the chromosome, of which 2849 protein-encoding genes were functionally assigned, with the remaining genes predicted as hypothetical proteins. We also found that 24 pseudogenes, six rRNA genes, 48 tRNA genes, and three other RNA genes that were distributed in the genome. The complete genome sequence of the PAMC28395 strain was deposited with an accession number of NZ_CP076547.1 in the NCBI database.

**Figure 1:**
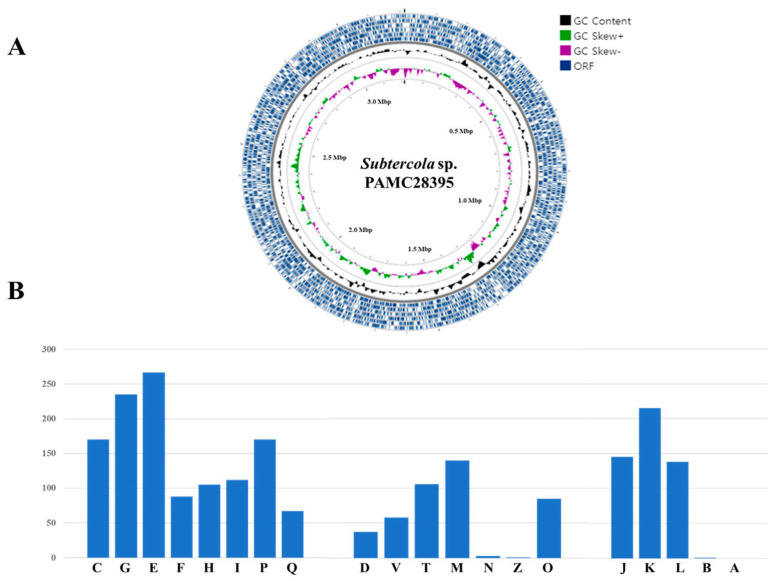
Circular map of *Subtercola* sp. PAMC28395’s genome (**A**) and Cluster of Orthologous Genes (COG) functional categories for forward coding sequences (**B**). Metabolism: C, energy production and conversion; G, carbohydrate transport and metabolism; E, amino acid transport and metabolism; F, nucleotide transport and metabolism; H, coenzyme transport and metabolism; I, lipid transport and metabolism; P, inorganic ion transport and metabolism; and Q, secondary metabolites biosynthesis, transport, and catabolism. Cell processing and signaling: D, cell cycle control, cell division, and chromosome partitioning; V, defense mechanisms; T, signal transduction mechanisms; M, cell wall/membrane/envelope biogenesis; N, cell motility; Z, mobilome, prophages, and transposons; and O, posttranslational modification, protein turnover, and chaperones. Information storage and processing: J, translation, ribosomal structure, and biogenesis; A, RNA processing and modification; K, transcription; L, replication, recombination, and repair; and B, chromatin structure and dynamics.

The circular genome map provided a comprehensive characterization of PAMC28395. In addition, we provided classification in 20 functional COG (Cluster of Orthologous Groups) categories (Figure 1). The most numerous COG categories are amino acid transport and metabolism (category E, 489 genes), carbohydrate transport and metabolism (category G, 235 genes), transcription (category K, 215 genes), energy production and conversion (category C, 170 genes), and inorganic ion transport and metabolism (category P, 170 genes), except for unknown function genes (category S, 489 genes) (Figure 1B). The fraction of proteins from a given genome assigned to particular COG functional categories is a helpful genome feature. It has been adopted by the Genome Standards Consortium as an essential characteristic of newly sequenced genomes [34]. Based on the COG annotation results, the predicted characteristics of the bacterial genome of PAMC28395 indicate the presence of the “metabolism” group, specifically falling between categories E and G. Category E corresponds to amino acid transport and metabolism, and category G pertains to carbohydrate transport and metabolism. This suggests that the gene content related to amino acid and carbohydrate metabolism is highly conserved within the genome of PAMC28395. In general, bacteria rely on cellular respiration to break down carbohydrates and obtain energy. This energy is then utilized in various cellular processes, including photosynthesis, where carbon dioxide is converted into carbohydrates. This process temporarily stores energy in high-energy molecules such as ATP (adenosine triphosphate). These metabolic mechanisms enable bacteria to adapt effectively to extreme environments [35,36]. We identified some genes related to amino acid transport, transcription, carbohydrate transport, and energy production or conversion. Thus, we predicted that this strain’s energy storage and carbohydrate metabolism functions would be higher than those of other bacteria strains because carbohydrates play a crucial role in many essential metabolic pathways.

### 3.2. 16S rRNA Phylogenetic Analysis and Calculation of ANI and dDDH

**Figure 2:**
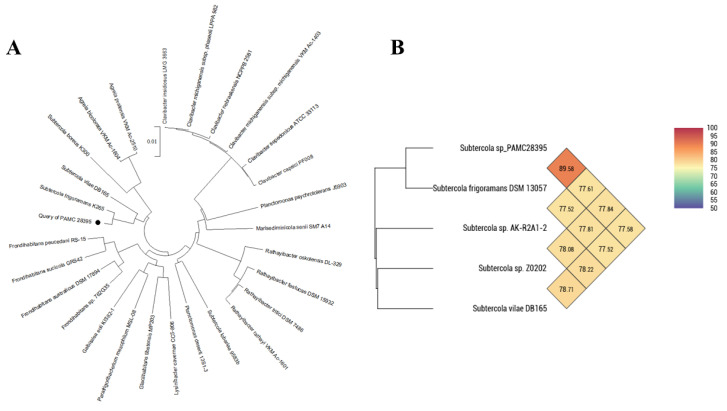
16S rRNA phylogenetic tree (**A**) and Orthologous Average Nucleotide Identity (ANI) (**B**) of *Subtercola* sp. PAMC28395. The phylogenetic tree was generated using the maximum likelihood method and Tamura–Nei model in MEGA X based on 16S rRNA sequences. In addition, all strains were calculated using an OrthoANI in OAT (Orthologous Average Nucleotide Identity Tool). Strains belonging to the same species are marked with a stronger color.

The identification of PAMC28395 was verified using 16S rRNA sequence analysis (Figure 2A). Further phylogenetic analysis of PAMC28395′s 16S rRNA sequence in comparison with those from some representative families within *Microbacteriaceae* (*Subtercola*, *Marisediminicola*, *Lysinibacter*, *Frondihabitans*, and *Glaciihabitans*) showed that the PAMC28395 strain was most closely related to *Subtercola* species. This strain was phylogenetically placed with S. *frigoramans* and S. *vilae*. Results from phylogenetic analysis, the Basic Local Alignment Search Tool, and EzBioCloud revealed that it was closely related to S. *frigoramans* K265^T^ (99.44%), S. *vilae* DB165^T^ (97.83%), and S. *lobariae* 9583b^T^ (96.87%). These results confirm that the PAMC28395 strain belongs to the family *Microbacteriaceae*, phylum *Actinobacteria*. However, strain S. *frigoramans* K265^T^ was reported based only on 16S rRNA sequence comparison. Since there were little chemical taxonomic and phenotypic data to support this, it was reported that strain K265 should be included in a genus as a new species, for which the name S. *frigoramans* gen. nov. was suggested [10]. Indeed, using a measure called “completeness” allows for a reliable calculation of sequence similarity between PCR-derived (amplified) sequences and genome-derived reference sequences for the 16S rRNA gene. EZBioCloud, a bioinformatics platform, suggests utilizing this measure for such calculations [37]. Based on the analysis conducted using EZBioCloud, it was observed that there are 94 gaps or differences in the 16S rRNA sequence between strain PAMC28395 (1531 bp sequenced from the genome) and S. *frigoramans* K265^T^ (1437 bp obtained through PCR). EZBioCloud calculated the similarity based on the completeness of the reference sequences. For instance, when the completeness of S. *frigoramans* K265^T^ is 99.3%, the calculated similarity with strain PAMC28395 is 99.44%. Similarly, when considering S. *vilae* DB165^T^ with a completeness of 100%, the calculated similarity is 97.83%. These results suggest that strain PAMC28395 likely belongs to the *Subtercola* genus and exhibits similarity to S. *frigoramans*. However, it is important to conduct further confirmatory experiments to provide more concrete evidence. Based on the observed differences and similarities, there is a possibility that PAMC28395 could be classified as a new species within the *Subtercola* genus, potentially positioned between S. *vilae* and S. *frigoramans*.

There is little genomic information on the *Subtercola* genus in the NCBI database. Not all strains mentioned above have whole genomes. Among registered strains in the NCBI database, only the two strains of PAMC28395 and AK-R2A1-2 have complete genome sequences among *Subtercola* species. However, strain AK-R2A1-2 has recently been identified as S. *endophyticus* by Jiang et al. [14]. These strains were chosen based on their whole genome sequences and placement within the same phylogenetic group in the 16S rRNA phylogenetic tree. Consequently, we compared average nucleotide identity (ANI) values between our strain and five strains with the sequenced genome data (Table S1). This comparison aimed to assess the ANI values and determine the bacteria species identified between our strain and the selected reference strains. In Figure 2B, the closest ANI value between PAMC28395 and the other type of strain was 89.58%, which was considerably lower than the threshold value of 96% for the boundary of species circumscription. ANI analysis shows the average nucleotide identity of all bacterial orthologous genes classified between any two genome sequences and offers identification between bacterial strains of the same or closely related species (i.e., species showing over 96% ANI values) [38,39]. The results presented in Supplementary Table S2 provide additional evidence suggesting that PAMC28395 exhibits digital DNA–DNA hybridization (dDDH) values (%) ranging from 17.9% to 60.7% when compared to the genomes of four other strains. Typically, genomes with dDDH > 70% are regarded as belonging to the same species. In addition, dDDH values ≤70% are commonly used as an indicator that the tested organism belongs to a different species than the reference strain [40]. However, the results clearly indicate that PAMC28395 is indeed a distinct species, differing from both S. *frigoramans* DSM13057 (60.7%) and S. *vilae* DB165 (18.7%). This conclusion is supported by the significantly lower values observed for PAMC28395, which fall below the 70% threshold. Therefore, both ANI and dDDH values support that PAMC28395 should be identified as a novel species between S. *vilae* and S. *frigoramans* in the *Subtercola* genus. Further clarification of the new species within the *Subtercola* genus would require additional experiments. By conducting comprehensive experiments such as phenotypic characterization, biochemical assays, growth conditions analysis, and additional genetic analysis, the potential new species’ unique characteristics and distinct traits can be elucidated, leading to more accurate classification and characterization. While phylogenetic analysis can provide valuable insights and comparing ANI and dDDH calculates the value, experimental validation is crucial for definitively establishing a new species. Thus, we have mentioned the strain as *Subtercola* sp. PAMC28395.

### 3.3. CAZyme-Encoding Genes in Subtercola sp. PAMC28395

CAZymes are carbohydrate-degrading enzymes that break down complex polysaccharides such as cellulose into simple sugars. This is crucial for the survival of bacteria. Through complete genome analysis of various strains and cold-adapted species, evolutionary insights can be revealed. For example, it has been reported that Bacillus sp. TK-2 exhibits adaptability to cold environments through the expression of CAZyme genes involved in the degradation of polysaccharides such as cellulose and hemicellulose [41]. However, the characteristics of glycogen metabolism in prokaryotes have not been well studied compared to eukaryotes. Particularly, the metabolism of microorganisms isolated from cold environments is not well known. This study predicted the role of CAZymes in cold adaptation and the characteristics of PAMC28395 involved in glycogen and trehalose metabolism.

**Figure 3:**
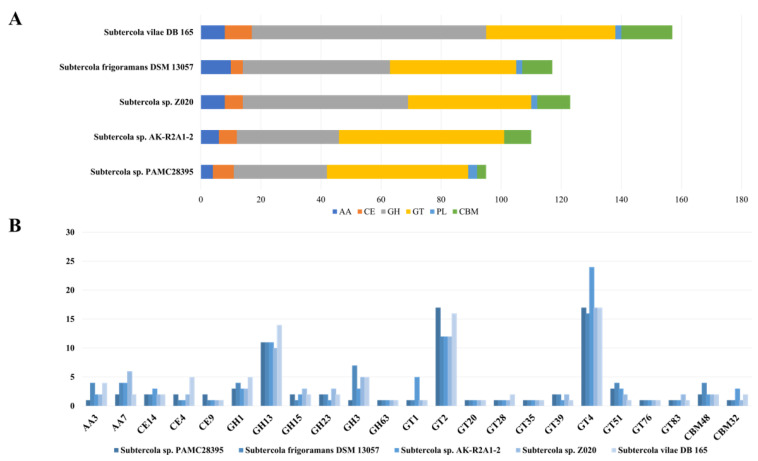
Comparative CAZyme-encoding genes found in the genome of *Subtercola* species (**A**) and the total CAZyme family of each strain. (**B**) The number of enzymes for each CAZyme family pattern they have in common: AA, auxiliary activity; CE, carbohydrate esterase; GH, glycoside hydrolase; GT, glycosyltransferase; PL, polysaccharide lyase; and CBM, carbohydrate-binding modules.

Since genes related to carbohydrate metabolism are expected to be highly conserved, we conducted a detailed analysis of CAZymes. Among a total of 2849 identified protein-encoding genes in strain PAMC28395, 92 were annotated and classified into the group of CAZymes (glycoside hydrolase, GH; glycosyltransferase, GT; carbohydrate esterase, CE; auxiliary activity, AA; polysaccharide lyase, PL; and carbohydrate-binding module, CBM) using dbCAN3. Results provided insight into the carbohydrate utilization mechanisms of strain PAMC28395. Using the Signal P tool, we predicted the retention of 12 genes encoding signal peptides in the CAZymes of strain PAMC28395. In addition, we anticipated that enzymes involved in carbohydrate metabolism would be distributed as follows: 31 GHs, 47 GTs, 7 CEs, 4 AAs, 3PLs, and 2 CBMs (Figure 3A).

GH gene annotation revealed that the PAMC28395 genome sequence contained genes involved in glycogen and trehalose metabolism pathways, such as β-glucosidase (GH1, EC3.2.1.21), 1,4-alpha-glucan (glycogen) branching enzyme (CBM48 and GH13_9, EC 2.4.1.18), malto-oligosyltrehalose synthase (GH13_26, EC 5.4.99.15), limit dextrin alpha-1,6-maltotetraose-hydrolase (GH13_11, EC 3.2.1.196), trehalose phosphorylase (GH65, EC 2.4.1.64), and malto-oligosyltrehalose trehalohydrolase (GH13_10, EC 3.2.1.141) (Table 2). Glycogen is a homopolysaccharide composed of alpha-D-glucose held together by alpha-1,4 and alpha-1,6 glycosidic bonds. It is mainly found in animals, fungi, and bacteria. It acts as a significant source of energy storage in bacteria [42]. Organisms in cold environments require efficient energy storage systems to adapt and survive. Glycogen synthesis is one such system, particularly in bacteria which employ a passive energy-saving strategy through slow glycogen degradation in nutrient-deprived conditions. Glycogen is hypothesized to function as a long durability energy reserve, which has been reported as a Durable Energy Storage Mechanism (DESM) to account for the long-term survival of some bacteria in cold environments [43]. In recent years, there have been numerous reports on the ability of microbes isolated from cold environments to adapt to and survive in these conditions, particularly concerning glycogen metabolism identified by genome analysis. For example, *Shigella* sp. strain PAMC28760, *Arthrobacter* sp. strain PAMC25564, and *Nakamurella* sp. strain PAMC28650 isolated from cold environments have been reported to be able to adapt and survive in cold environments through glycogen metabolism [44,45,46].

Moreover, we found interesting CAZyme-related genes in genome information. Microbial α-galactosidases (GH36) play a crucial role in the breakdown of plant-derived α-galactosidases by human gut microbiota. Here, we identified another GH36 gene, similar to *agaSK*. When expressed in vivo, it might contribute to α-galactosidase activity. Enzyme AgaSK isolated from *Ruminococcus gnavus* E1 (RG E1) has recently been characterized as a bifunctional enzyme composed of a GH36 α-galactosidase and a kinase domain that can phosphorylate sucrose provided by raffinose hydrolysis, highlighting a putative novel glycolytic pathway in bacteria for sucrose assimilation. It shows partial plant oligosaccharide-degrading abilities. It plays an essential role in metabolizing complex oligosaccharides, working in conjunction with other GH enzymes and contributing to the degradation of mixed fibers [47]. We also identified a gene of the GH92 family as a promising candidate for a novel and highly active bacterial alpha-1,2-mannosidase. Bacterial alpha-1,2-mannosidases are attractive enzymes for in vitro enzymatic glycan modification of high-mannose-type N-glycans in natural and recombinant glycoproteins to hybrid or complex-type N-glycans. They are also appealing targets for a better understanding of how commensals and other bacteria can utilize host glycoprotein glycans to shape the microbiota community [48]. Recently, it has been shown that some bacteria can use high-mannose-type N-glycans efficiently for growth but not sialylated N-linked glycans [49,50].

### 3.4. Comparison of CAZyme Patterns with Those from Closely Related Species

By revealing key CAZymes involved in the cold adaptation of *Subtercola* species, this study offers valuable insights into the evolutionary strategies of these microorganisms. It presents exciting opportunities for developing novel biotechnological tools with diverse industrial applications. Considering the accessibility of available genome data, complete genomes of five strains, including our strain, were chosen for comparative analysis of CAZymes. Our results showed that the number of total CAZymes in each genome ranged from a minimum of 95 (PAMC28395) to a maximum of 157 (*Subtercola* sp. DB165). Our genome sequence analysis of the five strains confirmed our predictions, revealing a consistent pattern of 23 CAZyme genes across all strains, including AA3, AA7, CE4, CE14, CE9, GH1, GH3, GH13, GH15, GH23, GH63, GT1, GT2, GT4, GT20, GT28, GT35, GT39, GT51, GT76, GT83, CBM32, and CBM48 (Figure 3B). Of particular note, CAZyme member GH13 associated with glycogen and trehalose metabolism and energy storage was identified in all strains, suggesting its fundamental role in the degradation of glycogen and trehalose pathways. The presence of a diverse family of CAZymes and related genes for proteins with a strong ability to store and release energy in *Subtercola* species isolated from extreme environments highlights their adaptive strategy for survival in a cold environment. Despite being a complete genome, PAMC28395 had the fewest number of CAZyme genes among the five strains analyzed. Although CAZymes are predominantly responsible for the degradation and biosynthesis/modification of polysaccharides, not all members of this protein group are secreted. Additionally, we identified minor differences in CAZyme gene patterns among *Subtercola* species. However, it is noteworthy that the two genes related to α-galactosidases (GH36) and bacterial alpha-1,2-mannosidase (GH92) had the highest probability of expression in this strain. In conclusion, our genomic comparison demonstrates the potential of these strains to degrade complex polysaccharides and store energy.

### 3.5. Glycogen/Trehalose and Pentose Phosphate Metabolic Pathway in Subtercola sp. PAMC28395

We investigated the glycogen/trehalose and pentose phosphate metabolic pathway to confirm related cold adaptation to a cold environment in PAMC28395 (Figures S1 and S2). This analysis assumed that the PAMC28395 strain would use glycogen/trehalose and pentose phosphate metabolic pathways to obtain energy or degrade polysaccharides. These strains showed related genes for one main pathway of trehalose biosynthesis and three pathways of producing D-glucose, which is responsible for maltose recycling to maltodextrins. In addition, we can predict that these enzymes constitute another method that bacteria use for sugar uptake when their energy source is phosphoenolpyruvate.

As a result, this strain probably produces polysaccharides by themselves and from an external source using phosphoenolpyruvate rather than consuming energy because they have a related recycling gene for degradation or production. To adapt and survive in a cold environment, organisms must have well-developed functional energy storage systems. Glycogen is one of the carbohydrates stored in animal cells. It is used as an energy reserve to adapt and survive in a cold environment. It has a three-dimensional molecular structure comprising the most representative α-1,4-glucoside linkage and α-1,6-glucosidase linkage among carbohydrates [51]. Enzymes involved in glycogen metabolism include glycogen synthase, glycogen phosphorylase, branching enzyme, debranching enzyme, and phosphatase. These enzymes belong to the GH 13 family of CAZymes. Understanding the regulation and function of these enzymes in glycogen metabolism is essential for comprehending the mechanisms underlying energy homeostasis and metabolic adaptation in response to cold exposure. The classical pathway (CP) of bacterial glycogen metabolism includes five essential enzymes: ADP-glucose pyrophosphorylase (GlgC), glycogen synthase (GlgA), glycogen branching enzyme (GlgB), glycogen phosphorylase (GlgP), and glycogen debranching enzyme (GlgX). GlgB degradation of α-1,6-linkages in the branch portion of the glycogen molecule is needed to assist GlgP in producing glucose molecules. Finally, phosphatase regulates the metabolic pathway by removing molecules containing phosphate groups generated during glycogen synthesis and breakdown processes [42,44].

Moreover, the pentose phosphate pathway (PPP) is a major pathway for glucose catabolism, along with glycolysis. However, the extent of its contribution to bacterial metabolic adaptation, particularly in the context of bacteria, remains largely unexplored. The PPP comprises two branches: an oxidative branch and a non-oxidative branch. Glucose flux through the oxidative branch generates NADPH, which is an essential reductant in anabolic processes. Meanwhile, the non-oxidative branch produces ribose-5P (R-5P) from glucose, which can be converted reversibly into glycolytic intermediates such as glyceraldehyde 3P (GA-3P) and fructose-6P (F-6P) [52,53]. Although a vast majority of bacteria isolated from extreme environments are capable of using C5 sugars as a carbon and energy source, the underlying metabolic pathways are not yet fully understood [54]. Genome sequence analysis of PAMC28395 and bioinformatic analysis have provided valuable insights into its metabolic capabilities and adaptability to cold environments. The strain utilizes the pentose phosphate pathway (PPP) for the degradation of C5 sugars and the production of pentose phosphates necessary for anabolic processes during glucose growth. Moreover, PAMC28395 possesses a range of carbohydrate-active enzymes and pathways associated with carbohydrate metabolism, enabling the degradation or production of various types of carbohydrates. These findings highlight the high adaptability of these strains to their environment, particularly cold environments. The presence of genes related to glycogen, trehalose, maltodextrin, and PPPs suggests their involvement in the degradation of polysaccharides, which may have implications for industrial applications. Microorganisms harboring these genes have the potential to produce D-glucose economically and harness their energy to thrive in cold environments. This adaptability is a characteristic often observed in bacteria thriving in extreme conditions such as cold environments. Furthermore, the results imply that the metabolism of trehalose in microorganisms depends on the specific metabolic requirements dictated by the environmental conditions they encounter.

### 3.6. Analysis of Secondary Metabolite BGCs and Antibiotic Resistance Genes

**Figure 4:**
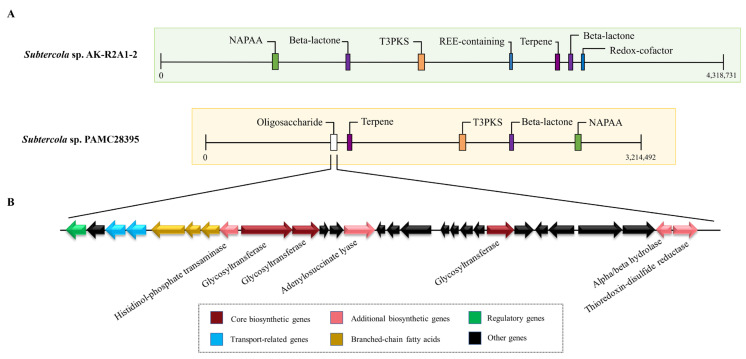
Schematic representation of the comparison between secondary metabolite biosynthetic gene clusters (BGCs). (**A**) Distribution of BGCs in *Subtercola* sp. PAMC28395 and *Subtercola* sp. AK-R2A1-2. (**B**) The putative oligosaccharide BGC of *Subtercola* sp. PAMC28395. Core biosynthetic genes and additional biosynthetic genes are highlighted in color with their predicted functions. Branched-chain fatty acid biosynthetic genes, transport genes, regulatory genes, and other genes are shown in yellow, blue, green, and black, respectively.

The production of bacterial secondary metabolites has also been proposed as a strategy for adaptation to extreme environments due to their potential diversity. Typically, secondary metabolites are synthesized through biosynthetic gene clusters (BGCs), which contain genes encoding core biosynthetic enzymes, transporters, and regulators found physically co-located. This knowledge drove us to investigate the genetic traits of the *Subtercola* genus evolutionarily, explicitly targeting those associated with cold environment adaptation to shed light on factors contributing to the bacterium’s diverse range of secondary metabolites. As shown in Table 3, antiSMASH analysis indicated that PAMC28395 had a total of five BGCs related to oligosaccharides, terpene, type III polyketide synthase (T3PKS), beta-lactone, and non-alpha poly-amino acids such as e-polylysin (NAPAA). Comparing putative BGCs in *Subtercola* sp. PAMC28395, *Subtercola* sp. AK-R2A1-2, *Subtercola* sp. Z020, S. *frigoramans* DSM13057, and S. *vilae* DB165 suggested that four clusters (terpene, T3PKS, beta-lactone, and NAPAA) were conserved with few differences (Tables S3 and S4, and Figures S3–S5). Except for AK-R2A1-2, none of the other strains had BGCs containing the redox cofactor (Figure 4A), which was thought to be associated with adaptation to low temperatures [55]. Instead, gene cluster-encoding branched-chain fatty acids/oligosaccharides were found in *Subtercola* sp. PAMC28395, *Subtercola* sp. Z020, S. *frigoramans* DSM13057, and S. *vilae* DB165, but not in *Subtercola* sp. AK-R2A1-2. In this gene region, while branched-chain fatty acid BGCs showed high similarity to known clusters, oligosaccharide BGCs containing three GTs were detected with low similarity to known clusters (Figure 4B). Considering that oligosaccharides are known to protect cell membranes through an ice inhibition effect [56,57], these biosynthetic genes may play an important role in the survival of those strains.

As an additional analysis for the characterization of BGCs, antibiotic resistance genes were identified with the ARTS tool [33]. This tool enables prioritization of BGCs corresponding to antibiotics based on the understanding that antibiotic producers harbor self-resistance genes within the same BGCs responsible for making antibiotics [58]. Comparing antibiotic resistance genes in *Subtercola* sp. PAMC28395, *Subtercola* sp. AK-R2A1-2, *Subtercola* sp. Z020, *S. frigoramans* DSM13057, and *S. vilae* DB165 revealed that strain PAMC28395 contained more core genes in BGCs than other strains (Table 4). Regarding the results of *Subtercola* sp. Z020 and *S. vilae* DB165, the number of core gene hits detected in a BGC was lower than the actual value due to analysis using a draft genome. Additionally, 19 resistance model genes were detected in PAMC28395, including dihydrolipoamide acetyltransferase, pyruvate carboxylase, pentapeptide repeat protein, DNA gyrases, DNA topoisomerases, and ornithine carbamoyltransferases (Table S5). In particular, the beta-lactone BGC described above contained pyruvate carboxylase, suggesting the possibility of producing inhibitors of this enzyme.

## 4. Conclusions

The complete genome sequence of PAMC28395 was elucidated in this study, and a comparative genome analysis with other species was conducted to investigate CAZyme patterns. This isolate was obtained from cryoconite under laboratory conditions. It was confirmed as a *Subtercola* species based on diverse phylogenetic methods. We found 95 active CAZyme genes, including four AAs, three CBMs, seven CEs, 31 GHs, 47 GTs, and three PLs of CAZyme families. Our predictions suggest that PAMC28395 can adapt quickly to its environment and produce energy autonomously. The genome size of the strain is 3.21 Mb, with a GC content of 64.5%. Glycogen and trehalose metabolism are associated with its CAZyme genes. In addition, two genes related to α-galactosidases (GH36) and bacterial alpha-1,2-mannosidase (GH92) had the highest probability of expression in this strain. Moreover, a comparative analysis of BGCs in several *Subtercola* strains, including PAMC28395, revealed they had oligosaccharide BGCs which might be associated with their adaptation to low temperatures. To the best of our knowledge, this is the first report of antibiotic resistance gene analysis in the *Subtercola* genus. Results of ARTS analysis revealed that multiple antibiotic resistance genes were present in all strains analyzed, which might suggest that they provide a complex self-resistance system for these strains to survive. Additional studies are needed to characterize these BGCs and determine their products and biological roles. In summary, genome sequence analysis can provide valuable information on novel functional enzymes, particularly CAZymes, that operate at low temperatures. The CAZymes can be used for biotechnological applications and fundamental research purposes. Moreover, this study forms a basis for understanding how the PAMC28395 strain produces energy in cold environments.

## Figures and Tables

**Table 1:** Genome features of *Subtercola* sp. PAMC28395.

Feature	Value
**A. Genome Statistics**	
Contig	1
Total length (bp)	3,214,492
N50	3,214,492
L50	1
GC content (%)	64.5
**B. Genome features**	
Assembly level	Complete genome
Genes	2930
Protein	2849
Pseudogenes	24
rRNA genes	6
tRNA genes	48

**Table 2:** Prediction results of the GH family of CAZymes from *Subtercola* sp. PAMC28395.

GH Family	Enzyme Activity	Gene Location	EC Number
GH1	Beta-glucosidase	275769_277226	EC 3.2.1.21
	Beta-glucosidase/beta-*D*-fucosidase	1505668_1507110	EC 3.2.1.21/ EC 3.2.1.38
	Beta-glucosidase	3028462_3026861	EC 3.2.1.21
GH3	Beta-*N*-acetylglucosaminidase	3132007_3131027	EC 3.2.1.52
GH5	Endoglucanase/endo-1,4-beta-glucanase	1292002_1293591	EC 3.2.1.4
	Endoglucanase/endo-1,4-beta-glucanase	283139_284821	EC 3.2.1.4
GH6	Beta-1,4-glucanase (cellulase)	1232268_1233245	EC 3.2.1.4
GH13	Malto-oligosyltrehalose trehalohydrolase	1915218_1913389	EC 3.2.1.141
	Limit dextrin alpha-1,6-maltotetraose-hydrolase	1877463_1875409	EC 3.2.1.196
	Limit dextrin alpha-1,6-maltotetraose-hydrolase	1919805_1917604	EC 3.2.1.196
	Limit dextrin alpha-1,6-maltotetraose-hydrolase	2317861_2319915	EC 3.2.1.196
	Trehalose synthase/alpha-amylase	2380696_2382417	EC 5.4.99.16/ EC 3.2.1.1
	Malto-oligosyltrehalose synthase	1917563_1915224	EC 5.4.99.15
	Glucanase glgE	1872324_1870189	EC 3.2.1.-
	Alpha-glucosidase	530778_532550	EC 3.2.1.20
	Oligo-1,6-glucosidase	767858_769579	EC 3.2.1.10
	Amylosucrase	2341070_2339187	EC 2.4.1.4
	1,4-Alpha-glucan (glycogen) branching enzyme	1870189_1867967	EC 2.4.1.18
GH14	Beta-amylase	2902484_2904043	EC3.2.1.2
GH15	Glucoamylase	3134358_3136247	EC 3.2.1.3
	Glucoamylase	2314490_2312595	EC 3.2.1.3
GH23	Peptidoglycan lyase	540715_539951	EC 4.2.2.n1
	Peptidoglycan lyase	2448999_2448115	EC 4.2.2.n1
GH25	LysM peptidoglycan-binding domain-containing protein	1327564_1326686	-
GH31	Alpha-xylosidase	1503329_1505671	EC 3.2.1.177
GH36	Alpha-galactosidase	1771245_1769200	EC 3.2.1.22
GH42	Beta-galactosidase	1776765_1774606	EC 3.2.1.23
GH63	Glucosidase	2557228_2560086	EC 3.2.1.20
GH65	Maltose phosphorylase/trehalose phosphorylase	1652990_1655497	EC 2.4.1.8/EC 2.4.1.64
GH76	Alpha-1,6-mannanase	3035588_3036697	EC 3.2.1.101
GH92	Alpha-1,2-mannosidase	2024160_2021845	EC3.2.1.130

**Table 3:** Putative BGCs identified by antiSMASH in the genome of *Subtercola* sp. PAMC28395.

Cluster	Type	From	To	Most Similar Known Cluster (% Gene Similarity)	MIBiG-ID *
Cluster 1	Oligosaccharide	923,143	955,149	Branched-chain fatty acids (100%)	BGC0001535
Cluster 2	Terpene	1,048,063	1,069,037	Carotenoid (25%)	BGC0000637
Cluster 3	T3PKS	1,878,107	1,919,117	Alkylresorcinol (100%)	BGC0000282
Cluster 4	Beta-lactone	2,244,583	2,270,325	Microansamycin (7%)	BGC0001666
Cluster 5	NAPAA	2,736,338	2,770,420	-	-

* The Minimum Information about a Biosynthetic Gene Cluster (Genomic Standards Consortium).

**Table 4:** Comparative analysis of antibiotic resistance genes in *Subtercola* sp. PAMC28395, *Subtercola* sp. AK-R2A1-2, *Subtercola* sp. Z020, *S. frigoramans* DSM13057, and *S. vilae* DB165 by ARTS.

Organisms	Total Genes	Total BGCs	Known Resistance	Core Genes	Gene Duplication	BGC Proximity	Phylogeny /HGT
*Subtercola* sp. PAMC28395	2874	5	19	440	12	35	165
*Subtercola* sp. AK-R2A1-2	3952	5	20	449	14	25	165
*Subtercola* sp. Z020	3453	5	21	434	12	16	140
*Subtercola frigoramans* DSM13057	3362	5	20	444	13	32	162
*Subtercola vilae* DB165	4013	4	20	444	19	18	182

Total genes, total genes detected; total BGCs, the number of biosynthetic clusters found with antiSMASH; known resistance, the numbers of resistance model hits; core genes, the number of core gene hits; gene duplication, the number of genes with a higher copy number; BGC proximity, the number of core gene hits in a BGC; and phylogeny/HGT, the number of core gene hits with incongruent phylogeny.

## Data Availability

Data is contained within the article or Supplementary Materials.

## Supplementary Materials

The following supporting information can be downloaded at: https://www.mdpi.com/article/10.3390/microorganisms11061480/s1.
