# Supplementary material for: Complete Genome Analysis of Subtercola sp. PAMC28395: Genomic Insights into Its Potential Role for Cold Adaptation and Biotechnological Applications
Source: Microorganisms. 2023 Jun 1;11(6):1480. doi: 10.3390/microorganisms11061480 (PMC10303725; doi:10.3390/microorganisms11061480)
Supplement: Supplementary file 1 [file microorganisms-11-01480-s001.zip › microorganisms-2420839-supplementary.pdf]

microorganisms

Supplementary Materials

Complete Genome Analysis of *Subtercola* sp. PAMC28395: Genomic Insights into Its Potential Role for Cold Adaptation and Biotechnological Applications

Ryoichi Yamada [1,2,†], So-Ra Han [1,2,3,†], Hyun Park [4] and Tae-Jin Oh [1,2,3,5,*]

[1] Department of Life Science and Biochemical Engineering, SunMoon University, Asan 31460, Republic of Korea; tarkahr825@sunmoon.ac.kr (R.Y.); 553sora@sunmoon.ac.kr (S.-R.H.)
[2] Bio Big Data-Based Chungnam Smart Clean Research Leader Training Program, SunMoon University, Asan 31460, Republic of Korea
[3] Genome-Based BioIT Convergence Institute, Asan 31460, Republic of Korea
[4] Division of Biotechnology, College of Life Science and Biotechnology, Korea University, Seoul 02841, Republic of Korea; hpark@korea.ac.kr
[5] Department of Pharmaceutical Engineering and Biotechnology, SunMoon University, Asan 31460, Republic of Korea
* Correspondence: tjoh3782@sunmoon.ac.kr
† These authors contributed equally to this work.

Supplementary Tables

Table S1. Genomic information list for comparative genomic.

Strains	Features	Values
Subtercola sp. AK-R2A1-2 (Accession number: NZ_CP087997.1)	A. Genome Statistics	
	Contig	1
	Total length (Mb)	4.31873
	N50	4,318,731
	L50	1
	GC content (%)	65.8
	B. Genome features	
	Assembly level	Complete genome
	Genes	4,001
	Protein	3,874
	Pseudogenes	71
	rRNA genes	5
	tRNA genes	47
Subtercola sp. Z020 (Accession number: NZ_CP087997.1)	A. Genome Statistics	
	Contig	167
	Total length (Mb)	3.53326
	N50	36,800
	L50	27
	GC content (%)	69.0

	B. Genome features	
	Assembly level	Scaffold
	Genes	3,399
	Protein	3,292
	Pseudogenes	54
	rRNA genes	5
	tRNA genes	45
Subtercola sp. Z020 (Accession number: NZ_PSTT00000000.1)	A. Genome Statistics	
	Contig	167
	Total length (Mb)	3.53326
	N50	36,800
	L50	27
	GC content (%)	69.0
	B. Genome features	
	Assembly level	Scaffold
	Genes	3,399
	Protein	3,292
	Pseudogenes	54
	rRNA genes	5
	tRNA genes	45
Subtercola frigoramans DSM13057 (Accession number: NZ_JAFBBU000000000.1)	A. Genome Statistics	
	Contig	1
	Total length (Mb)	3.6796
	N50	3,679,597
	L50	1
	GC content (%)	64.4
	B. Genome features	
	Assembly level	Contig
	Genes	3,398
	Protein	3,155
	Pseudogenes	189
	rRNA genes	6
	tRNA genes	45
Subtercola vilae DB165 (Accession number:)	A. Genome Statistics	
	Contig	103
	Total length (Mb)	4.04314
	N50	87,665
	L50	15
	GC content (%)	65.1
	B. Genome features	
	Assembly level	Contig

Genes	3,836
Protein	3,691
Pseudogenes	89
rRNA genes	6
tRNA genes	47

Table S2. Summary of results found in the analysis of dDDH.

**Query genome**	**Reference genome**	**DDH**	**Model C.I.**	**Distance**	**Prob. DDH >= 70%**	**G+C difference**
Subtercola sp. PAMC28395	*Subtercola frigoramans* DSM13057	60.7	[57.0 - 64.3%]	0.2357	44.58	0.03
Subtercola sp. PAMC28395	*Subtercola* sp. AK-R2A1-2	18.0	[15.0 - 21.6%]	0.7689	0	1.30
Subtercola sp. PAMC28395	*Subtercola* sp. Z0202	18.8	[15.7 - 22.4%]	0.7449	0	4.49
Subtercola sp. PAMC28395	*Subtercola vilae* DB165	18.7	[15.6 - 22.2%]	0.7493	0	0.65
Subtercola frigoramans DSM13057	*Subtercola* sp. AK-R2A1-2	17.9	[14.8 - 21.5%]	0.7726	0	1.33
Subtercola frigoramans DSM13057	*Subtercola* sp. Z0202	18.6	[15.5 - 22.2%]	0.7509	0	4.52
Subtercola frigoramans DSM13057	*Subtercola vilae* DB165	18.2	[15.1 - 21.8%]	0.7636	0	0.68
Subtercola sp. AK-R2A1-2	*Subtercola* sp. Z0202	18.9	[15.8 - 22.5%]	0.7429	0	3.19
Subtercola sp. AK-R2A1-2	*Subtercola vilae* DB165	19.3	[16.2 - 22.9%]	0.7310	0	0.65
Subtercola sp. Z0202	*Subtercola vilae* DB165	20.6	[17.4 - 24.3%]	0.6950	0	3.84

Table S3. Putative BGCs identified by antiSMASH in the genome of *Subtercola* sp. AK-R2A1-2.

Cluster	Type	From	To	Most similar known cluster (% gene similarity)	MIBiG-ID*
Cluster 1	NAPAA	940,093	974,349	-	-
Cluster 2	Beta-lactone	1,478,451	1,502,396	Microansamycin (7%)	BGC0001666
Cluster 3	T3PKS	2,015,037	2,056,128	Alkylresorcinol (66%)	BGC0000282
Cluster 4	RRE-containing	2,692,066	2,709,921	Kosinostatin (3%)	BGC0001073
Cluster 5	Beta-lactone	3,035,049	3,068,677	-	-
Cluster 6	Terpene	3,068,677	3,156,939	Carotenoid (50%)	BGC0000644
Cluster 7	Redox-cofactor	3,218,696	3,243,543	Lipopolysaccharide (5%)	BGC0000774

* The Minimum Information about a Biosynthetic Gene Cluster (Genomic Standards Consortium).

Table S4. Putative BGCs identified by antiSMASH in the genome of *Subtercola frigoramans* DSM13057.

Cluster	Type	From	To	Most similar known cluster (% gene similarity)	MIBiG-ID*
Cluster 1	Oligosaccharide	19,634	51,740	Branched-chain fatty acids (100%)	BGC0001535
Cluster 2	NAPAA	1,654,358	1,688,425	-	-
Cluster 3	Beta-lactone	2,162,606	2,188,417	Microansamycin (7%)	BGC0001666
Cluster 4	T3PKS	2,585,087	2,626,178	Alkylresorcinol (100%)	BGC0000282
Cluster 5	Terpene	3,501,426	3,522,400	Carotenoid (25%)	BGC0000637

* The Minimum Information about a Biosynthetic Gene Cluster (Genomic Standards Consortium).

Table S5. Known antibiotic resistance genes in the genome of *Subtercola* sp. PAMC28395 detected by ARTS analysis.

Model	Description	Sequence ID	E-value	Bit-score	Gene Location
RF0007	ABC_efflux	109	4.8E-75	250.5	110923_112741
RF0007	ABC_efflux	123	6.1E-88	293.1	129666_131550
TIGR02013	TIGR02013	218	0	1717.4	220667_224159
PF00364.17	Biotin_lipoyl	765	4.7E-16	55.9	805997_807431

PF13599.1	Pentapeptide_4	842	7.6E-13	45.8	900949_901621
**PF00364.17**	**Biotin_lipoyl**	**875**	**2.2E-21**	**72.9**	**928218_929700**
TIGR00663	TIGR00663	911	1.1E-79	265.4	964984_966130
PF00364.17	DNA_gyraseB	915	6.3E-62	205.5	968963_970925
PF00521.15	DNA_topoisoIV	916	7.2E-140	464.2	971052_973620
PF00364.17	Biotin_lipoyl	1292	2E-17	60.3	1412950_1414750
PF01039.17	Carboxyl_trans	1299	2.1E-194	644.1	1421393_1422965
PF00185.19	OTCace	1949	1.2E-52	175.6	2142886_2143822
**PF00364.17**	**Biotin_lipoyl**	**2054**	**1.1E-14**	**51.5**	**2254582_2257981**
PF00044.19	Gp_dh_N	2201	3.6E-58	193.4	2411619_2412627
PF00185.19	OTCace	2264	3.7E-34	115.5	2494806_2495763
PF00521.15	DNA_topoisoIV	2343	9.9E-139	460.4	2586155_2588645
PF00204.20	DNA_gyraseB	2345	1.1E-43	146.1	2589241_2591323
PF00364.17	Biotin_lipoyl	2354	9.7E-26	86.9	2602407_2603847
RF0003	AAC3-I	2769	3.5E-23	79.9	3075622_3076078

Highlighted in bold are the antibiotic resistance genes detected in the BGCs. Sequence IDs 875 and 2054 are located in the oligosaccharide synthetic gene cluster and beta-lactone synthetic gene cluster, respectively.

Supplementary Figures

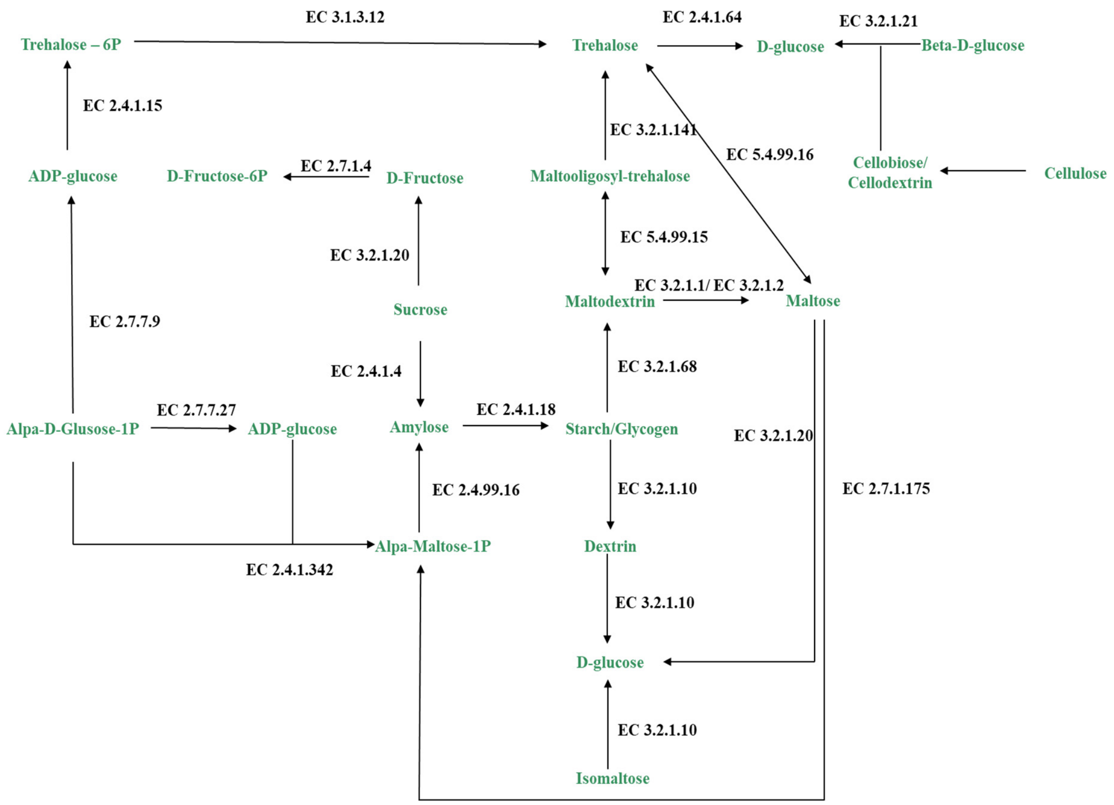

Figure S1. Predicted pathways for glycogen and trehalose metabolism in *Subtercola* sp. PAMC28395 as cold adaptation response.

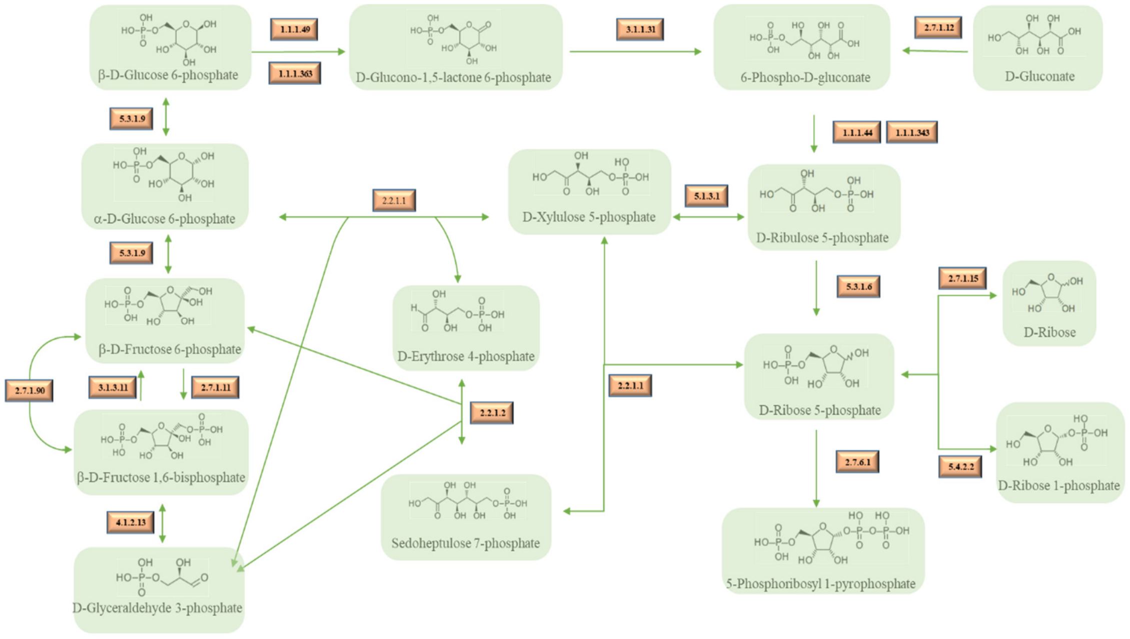

Figure S2. Predicted pathways for pentose phosphate pathway in *Subtercola* sp. PAMC28395 as cold adaptation response.

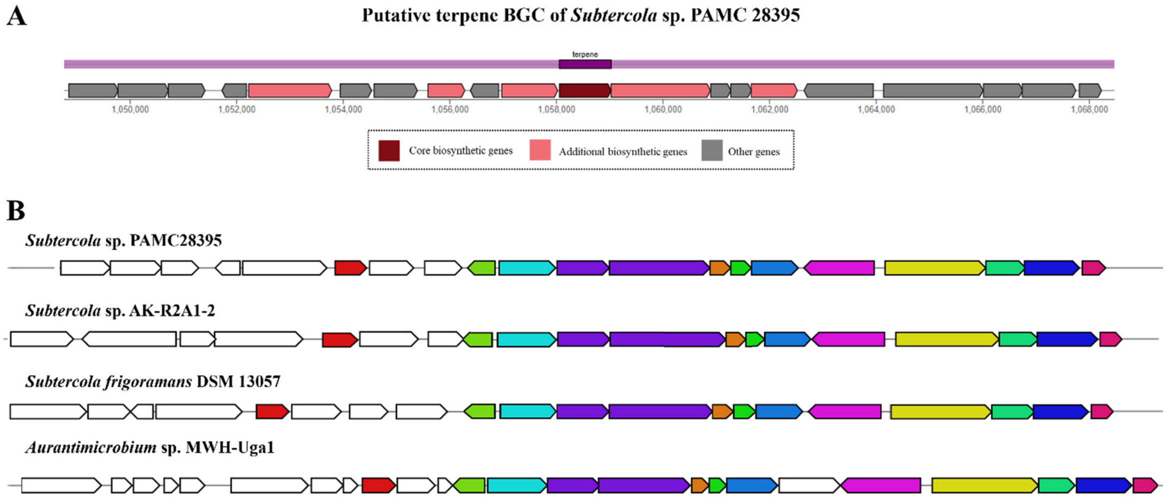

Figure S3. Summary of putative terpene BGCs. (A) Terpene BGCs of *Subtercola* sp. PAMC28395 detected by antiSMASH. (B) Comparison of *S.* sp. PAMC28395, *S.* sp. AK-R2A1-2, and *S. frigoramans* DSM13057. The terpene cluster from *Aurantimicrobium* sp. MWH-Uga1, the known most similar, is also described. Colors are matched to shared genes and differences in sequence similarity are shown according to reported data (Biosample data from NCBI database).

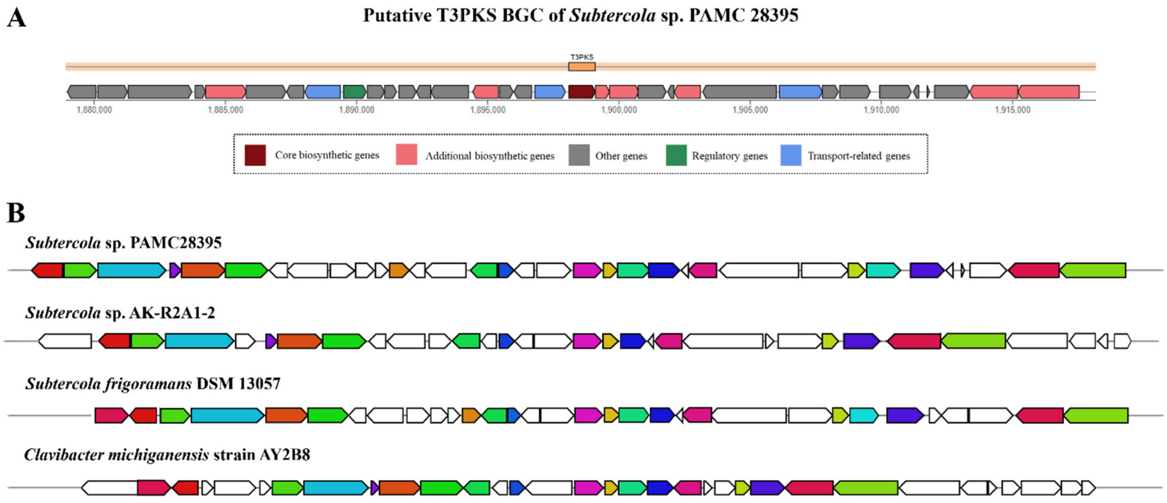

Figure S4. Summary of putative T3PKS BGCs. (A) T3PKS BGCs of *Subtercola* sp. PAMC28395 detected by antiSMASH. (B) Comparison of *S.* sp. PAMC28395, *S.* sp. AK-R2A1-2, and *S. frigoramans* DSM13057. The T3PKS cluster from *Clavibacter michiganensis* strain AY2B8, the known most similar, is also described. Colors are matched to shared genes and differences in sequence similarity are shown according to reported data (Biosample data from NCBI database).

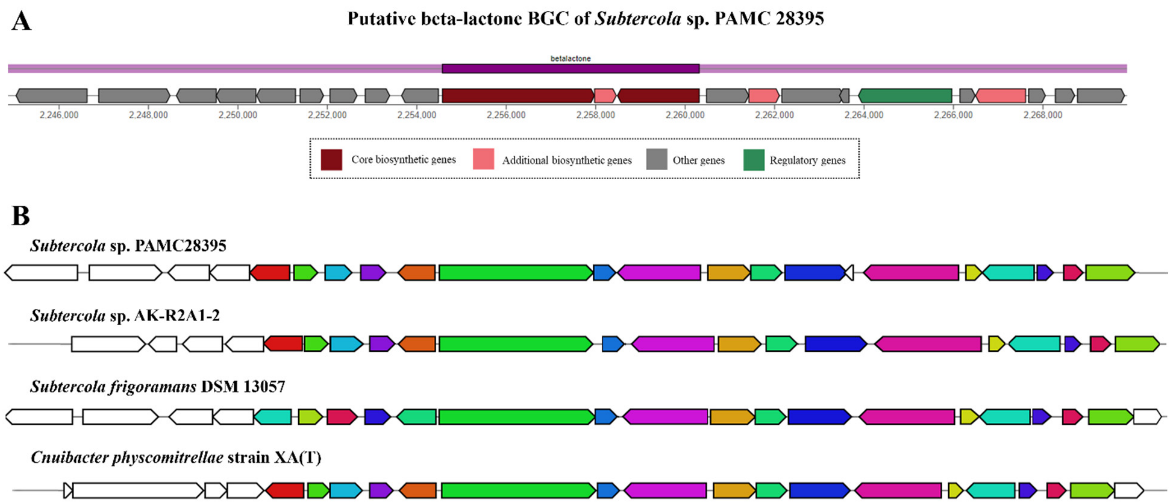

Figure S5. Summary of putative beta-lactone BGCs. (A) Beta-lactone BGCs of *Subtercola* sp. PAMC28395 detected by antiSMASH. (B) Comparison of *S.* sp. PAMC28395, *S.* sp. AK-R2A1-2, and *S. frigoramans* DSM13057. The beta-lactone cluster from *Cnuibacter physcomitrellae* strain XA(T), the known most similar, is also described. Colors are matched to shared genes and differences in sequence similarity are shown according to reported data (Biosample data from NCBI database).

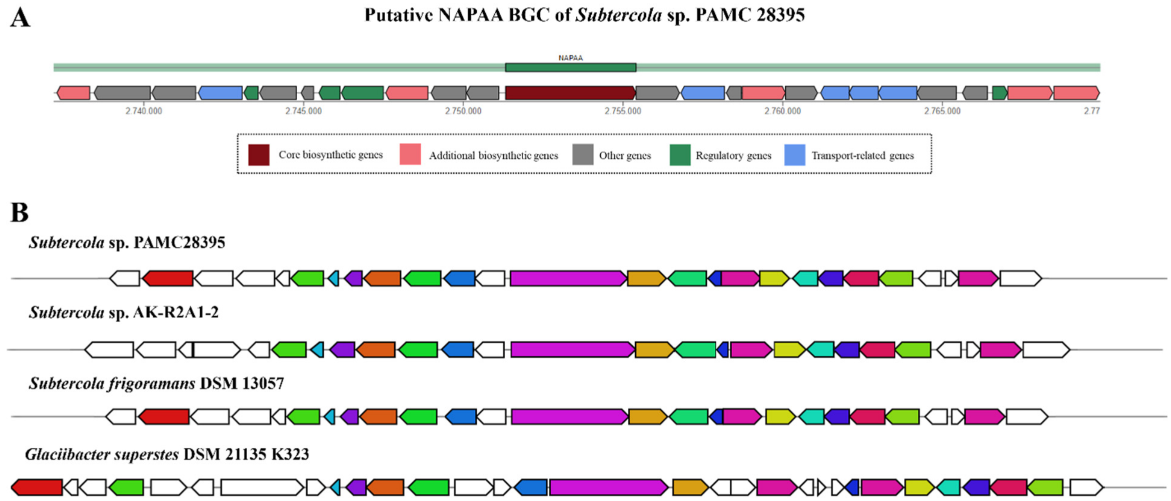

Figure S6. Summary of putative NAPAA BGCs. (A) NAPAA BGCs of *Subtercola* sp. PAMC28395 detected by antiSMASH. (B) Comparison of *S.* sp. PAMC28395, *S.* sp. AK-R2A1-2, and *S. frigoramans* DSM13057. The NAPAA cluster from *Glaciibacter superstes* DSM21135 K323, the known most similar, is also described. Colors are matched to shared genes and differences in sequence similarity are shown according to reported data (Biosample data from NCBI database).
